# Correlation of Fasting Lipid Profile in Patients With Chronic Liver Disease: A Descriptive Cross-Sectional Study in Tertiary Care Hospital

**DOI:** 10.7759/cureus.11019

**Published:** 2020-10-18

**Authors:** Abubakar Tauseef, Maryam Zafar, Behzad Rashid, Joseph Thirumalareddy, Victor Chalfant, Umar Farooque, Mohsin Mirza

**Affiliations:** 1 Internal Medicine, Creighton University School of Medicine, Omaha, USA; 2 Internal Medicine, Dow International Medical College, Dow University Hospital, Dow University of Health Sciences, Karachi, PAK; 3 Hospital Medicine, Creighton University School of Medicine, Omaha, USA; 4 Neurology, Dow International Medical College, Dow University Hospital, Dow University of Health Sciences, Karachi, PAK

**Keywords:** correlation, lipid panel, chronic liver disease, descriptive, tertiary care

## Abstract

Introduction: Chronic liver disease (CLD) is a term used to describe a wide spectrum of disorders, including idiopathic, infectious, genetic, drug-induced, toxin-induced, and autoimmune disorders. The common consequence of chronic damage to the liver is cirrhosis. Cirrhotic patients are further classified by their severity based on the Child-Pugh scoring system. Currently, Child-Pugh scoring consists of ascites, hepatic encephalopathy (HE), prothrombin time, serum albumin level, and total bilirubin level. Lipid panel in CLD is a great marker in determining the severity of CLD.

Method and methodology: It was a descriptive cross-sectional study conducted at a tertiary care hospital. A sample size of 122 was calculated by using a RaoSoft Digital Sample Size Calculator (RaoSoft, Inc., Seattle, WA) in which we used 5% as a margin of error, 95% as confidence interval (CI), 178 as population size, and response distribution as 50%. Non-complicated CLD patients having age in between 15 and 80 years with no cirrhotic complications including HE, spontaneous bacterial peritonitis, hepato-pumonary, or hepato-renal syndrome were included in our study; the rest of the CLD patients were excluded from our study.

Results: The mean age of the study population was 47.09 ± 12.30 years with more than half of the patients lying among the age group 25-50 years. The study population included 76% of males (n=93) and 24% of females (n=29), with a mean age of females higher than the males. Diabetes mellitus (58.19%) was the most frequent comorbidity associated with CLD in subjects included in our study. Parameters of lipid panel were decreased exponentially as the severity of CLD increases from Child score A to C. Total cholesterol, low-density lipoprotein (LDL), very-low-density lipoprotein (VLDL), high-density lipoprotein (HDL), and triglyceride (TG) level decreased as the severity increases in our study. The mean model for end-stage liver disease (MELD) score increased as per hypothesized as the severity increases from Child score A to Child score C, respectively.

Conclusion: Our study concluded that as the severity of CLD increases from Child class A to Child class C, the lipid panel profile decreases exponentially which proved the idea that had been hypothesized at the beginning of our study.

## Introduction

Chronic liver disease (CLD) is a term used to describe a wide spectrum of disorders, including idiopathic, infectious, genetic, drug-induced, toxin-induced, and autoimmune disorders [1,2]. The common consequence of chronic damage to the liver is cirrhosis [3]. This is characterized by the replacement of normal liver tissue by fibrotic tissue, occurring due to the accumulation of extracellular material, such as type I collagen activated by hepatic stellate cells and myofibroblasts [2]. The result is progressive liver dysfunction and clinical complications including portal hypertension (HTN), hepatocellular carcinoma (HCC), liver failure which is characterized by decompensation events such as ascites, variceal bleeding, encephalopathy, spontaneous bacterial peritonitis, and hepato-renal syndrome, eventually leading to death [3,4]. CLD is responsible for approximately 2 million deaths per year worldwide. Most deaths occur due to the complications of cirrhosis and HCC, making them the 11th and 16th most common causes of death, respectively [5]. The main contributing elements to CLD in Western and industrialized countries are alcohol and non-alcoholic fatty liver disease (NAFLD), whereas, in Asian countries, infectious causes like hepatitis B virus (HBV) and hepatitis C virus (HCV) are prominent causes [5,6]. The prevalence of HBV infection in northern Pakistan is 2.5%, while HCV continues to be the most important cause of HCC and CLD in the region with a prevalence of 4.5-8% [6].

Cirrhotic patients require multiple hospitalizations for their management. Therefore, choosing the proper treatment plan depends on the severity of cirrhosis, which can be evaluated by the Child-Turcotte-Pugh criteria [7]. The Child-Pugh score was initially proposed for assessment of the operative risk in patients presenting with variceal bleed. It primarily comprised of ascites, hepatic encephalopathy (HE), nutritional status, total bilirubin, and albumin. It was later modified with the replacement of nutritional status with prothrombin time or an international normalized ratio (INR). This model has been extensively used to evaluate the severity of liver dysfunction in clinical practice [8]. Chronic liver failure is correlated with a poor prognosis, characterized by early decompensation (Child-Pugh class B) and advanced decompensation (Child-Pugh class C), which have a two-year survival rate of 60% and 35%, respectively [4].

Lipids are an essential form of energy storage and comprise of fatty acids and their derivatives (tri-, di-, monoglycerides, and phospholipids, along with other sterol containing compounds such as cholesterol) [9]. The lipoprotein particles comprise of nascent high-density lipoproteins (HDLs) and very-low-density lipoproteins (VLDLs), which are synthesized and secreted into the circulation by the liver, while mature particles such as low-density lipoproteins (LDLs), intermediate-density lipoproteins, chylomicron remnants, and HDL are taken up by the liver in a regulated, receptor-dependent manner [10]. The liver plays a vital role in de novo cholesterol synthesis; therefore, an abnormal concentration is commonly found in patients with CLD [10,11].

A prominent decline has been documented in plasma cholesterol and triglyceride (TG) levels (i.e., from 166.5 to 121.2 mg/dl in regard to cholesterol levels and from 122 mg/dl to 92 mg/dl in regard to TG levels as the severity worsens from classes A to C) in patients with CLD due to the impairment in lipoprotein biosynthesis [7]. Furthermore, it has been demonstrated that high-density lipoprotein cholesterol (HDL-C) is closely correlated with liver function and an independent predictor of transplant-free mortality in cirrhotic patients [10].

The primary aim of this study was to determine and correlate the patterns of fasting lipid profile in patients, with varying degrees of severity in CLD. We conducted a cross-sectional study as it enabled us to assess our series of cirrhotic patients newly diagnosed at our outpatient clinics, hence showing the utmost recent information on the effect of lipid profile on CLD.

## Materials and methods

This study aimed to compare the determinants of lipid panel among the patients of CLD, conducted as a descriptive, cross-sectional study involving patients visiting the gastroenterology clinic at Tertiary care hospital between July 2019 and June 2020. All patients who met the inclusion criteria were followed with the reports of fasting lipid profiles which were correlated with the severity of the CLD.

This study included all those patients newly diagnosed with CLD defined by a radiological and clinical assessment with informed consent. The age group included was between 15 and 80 years. The duration of the diagnosis was set within six months before inclusion in the study. The study excluded all those patients who were not fulfilling the criteria of CLD or showing only hepatic steatosis (echogenicity) on liver ultrasound without fibrosis. Patients having complications of CLD including, variceal bleeding and hepatorenal syndrome were excluded from our study. A total of 122 patients met the inclusion criteria, and the sampling was done via non-probability consecutive methods.

All analysis was conducted by using the IBM SPSS version 25.0 (IBM Corp., Armonk, NY). A sample size of 122 was calculated by using a Raosoft Digital Sample Size Calculator (RaoSoft, Inc., Seattle, WA; http://www.raosoft.com/samplesize.html) in which we used 5% as a margin of error, 95% as confidence interval (CI), 178 as population size, and response distribution as 50%. All continuous variables were described as a mean and standard deviation which were then compared using independent sample t-test and one-way ANOVA. The comparison of categorical data was done either using the Chi-square test or Fisher’s exact test, depending on the limitation of data among the groups. A p-value of <0.05 was considered statistically significant (two-tailed).

## Results

The mean age of the study population was 47.09 ± 12.30 with more than half of the patients lying among the age group 25-50 years. The study population included 76% males (n=93) and 24% females (n=29), with a mean age of females higher than the males. The frequent comorbidities (other than CLD) were diabetes in 58.19% of the population, HTN in 27.86% of the population, ischemic heart disease in 10.65% of the population, and chronic kidney disease (CKD) in 9.83% of the population, respectively, while 32 patients were suffering from viral hepatitis, yet the most common cause of CLD found in our study was due to NAFLD.

According to the Child-Pugh classification of CLD, 65 (53.27%) patients belonged to class A, 43 patients (35.24%) to class B, and 14 patients (11.47%) to class C. Strikingly, 27 patients (22.13%) patients presented without other comorbidities yet had NAFLD leading to CLD. Among the 65 subjects included in Child’s class A, 40 (78.4%) subjects had a total cholesterol of >200 mg/dl, 36 (55.4%) subjects had LDL of 130 mg/dl, 47 (72.3%) subjects had a HDL level of <40 mg/dl, 31 (47.7%) subjects had a TG level of >150 mg/dl and VLDL level of >30 mg/dl, and 7 (10.8%) subjects had a mean MELD score of >9, respectively (Table 1).

**Table 1:** Correlation of fasting lipid profile among the Child-Pugh’s classification. LDL: low-density lipoprotein, HDL: high-density lipoprotein, VLDL: very-low-density lipoprotein, MEDL: model for end-stage liver disease, TG: triglycerides.

#	Laboratory investigations	Class A (n=65)	Class B (n=43)	Class C (n=14)	p-value
1	Total cholesterol	212.89 ± 38.29	169.16 ± 46.43	150.42 ± 28.76	<0.001
	>200 mg/dl	n=40 (78.4%)	n=11 (21.6%)	n=0 (0.0%)	<0.001
2	LDL	138.92 ± 33.34	100.65 ± 43.50	79.00 ± 23.97	<0.001
	>130 mg/dl	n=36 (55.4%)	n=5 (11.6%)	n=0 (0.0%)	<0.001
3	HDL	38.13 ± 13.85	33.20 ± 11.00	40.35 ± 13.39	0.082
	<40 mg/dl	n=47 (72.3%)	n=33 (76.7%)	n=5 (35.7%)	0.019
4	TG	172.75 ± 97.75	137.23 ± 84.34	105.85 ± 50.30	0.017
	>150 mg/dl	n=31 (47.7%)	n=13 (30.2%)	n=2 (14.3%)	0.029
5	VLDL	34.64 ± 19.57	27.37 ± 16.88	21.14 ± 10.08	0.015
	>30 mg/dl	n=31 (47.7%)	n=13 (30.2%)	n=2 (14.3%)	0.029
6	Cholesterol/HDL ratio	6.12 ± 2.13	5.57 ± 2.20	3.62 ± 1.22	<0.001
	>5	n=43 (66.2%)	n=17 (39.5%)	n=2 (14.3%)	<0.001
7	LDL/HDL ratio	4.03 ± 1.72	3.14 ± 1.28	2.00 ± 1.20	<0.001
	>2.5	n=55 (84.6%)	n=31 (72.1%)	n=2 (14.3%)	<0.001
8	TG/HDL ratio	5.48 ± 4.87	4.91 ± 4.53	2.52 ± 1.10	0.085
	>3	n=45 (69.2%)	n=29 (67.4%)	n=4 (28.6%)	0.013
9	Mean MELD score	8.09 ± 3.30	13.88 ± 6.32	23.57 ± 7.93	<0.001
	>9	n=7 (10.8%)	n=28 (65.1%)	n=14 (100.0%)	<0.001

Forty three subjects belonged to Child’s class B, out of which 11 (21.6%) subjects had a total cholesterol of >200 mg/dl, 05 (11.6%) subjects had LDL of 130 mg/dl, 36 (6.7%) subjects had a HDL level of < 40 mg/dl, 13 (30.2%) subjects had a TG level of >150 mg/dl and VLDL level of >30 mg/dl, 28 ( 65.1%) subjects had a mean MELD score of >9 (Table 1). The remaining 14 subjects were having a very progressed CLD, so they were classified in Child class C, out of those 14 subjects, not even a single subject had a total cholesterol of >200 mg/dl, the same goes for LDL of > 130 mg/dl, five (35.7%) subjects had an HDL level of < 40 mg/dl, two (41.3%) subjects had a TG level of >150 mg/dl and VLDL level of >30 mg/dl. Interestingly, all the subjects had a mean MELD score of >9 which has been hypothesized at the beginning of this study (Table 1).

## Discussion

In our study, the age of individuals suffering from CLD ranged from 25 to 50 years with a mean age of 47 years rendering it a disease of middle age coinciding with results of multiple studies [12-16], while our results also contrasted with few studies [17-20]. In our study, males were significantly more affected than females proving genetic predilection toward gender, results coinciding with outcomes of numerous studies [12,14,15,17,18,20], while opposing with outcomes of countable studies [13,16,19]. The most prevalent etiology leading to the development of cirrhosis in our study was a NAFLD followed by HCV and HBV, synchronizing with outcomes of negligible studies [16,20] while contrasting with findings of various studies [12-15,17,20]. In our study, the most common comorbidities suffered by individuals were diabetes mellitus followed by HTN and CKD results correlating with few studies [3,16].

In our study, most individuals belonging to non-HBV and HCV associated cirrhosis as compared to cirrhosis associated with HBV and HCV, an outcome coinciding with the results of a few studies [16,20]. Patients suffering from cirrhosis not associated with HBV and HCV had more elevated levels of alanine aminotransferase, outcome correlating with a couple of studies [12,16,20]. Serum fasting lipid profile comprising of total cholesterol, VLDL, HDL, and TG levels were slightly decreased in individuals belonging to our study suffering from cirrhosis associated with HBV and HCV outcome coinciding with the results of numerous studies [7,13,15,17,21]. Levels of serum lipid profile in isolated cases of NAFLD is usually elevated [16,20]. In our study, various ratios of serum lipid profiles such as HDL/LDL, TG/HDL, and TC/HDL are also declined with a prominent decrease in LDL/HDL and TG/HDL ratios in individuals suffering from HBV- and HCV-associated cirrhosis finding consistency with the outcome of one study [13].

In our study, levels of serum lipid profile including total cholesterol, total TG, VLDLs have their levels declining as the disease progressed from Child-Pugh A to C, finding consistency with many studies [13,15,17,21], while levels of LDL also declined as the disease progressed but another study conducted in similar pattern quoted elevating levels of LDL thus contrasting our results [18]. In our study, levels of HDL had intermediate raise in Child-Pugh class A with decline recorded in Child-Pugh B and again raised in Child-Pugh C, an outcome contrasting with the findings of numerous studies [3,7,13,17,18]. In our study, HDL had no role in determining the severity of the disease, an outcome contrasting with numerous studies [15,17,18,21].

## Conclusions

All the lipid markers were significantly higher in Child-Pugh A but starts declining with the increasing severity of CLD. Other than HDL, which was found not correlating with the severity of the liver disease, it declines in Child class B but again shown incremental response in class C. Among the ratios, LDL/HDL was the sensitive marker in determining the severity of the disease. It is therefore concluded that the amount of decrements measured in the levels of serum total cholesterol, LDL, and HDL in patients with cirrhosis are related to the progress in cirrhosis. Further studies are needed to assess the predictive values of measuring lipid profiles as a means to estimate the extent of liver damage in cirrhotic patients.
